# The temperature sensitivity of motor units in rat soleus

**DOI:** 10.1038/s41598-024-53208-8

**Published:** 2024-02-06

**Authors:** Bartosz Malak, Jan Celichowski, Hanna Drzymała-Celichowska

**Affiliations:** 1 Department of Neurobiology, Poznan University of Physical Education, 27/39 Królowej Jadwigi St., 61-871 Poznań, Poland; 2 Department of Physiology and Biochemistry, Poznan University of Physical Education, 27/39 Królowej Jadwigi St., 61-871 Poznań, Poland

**Keywords:** Neuroscience, Physiology

## Abstract

Temperature has a significant impact on the performance of the neuromuscular system and motor control processes. The smallest functional components of these systems are motor units (MUs), which may differ significantly between different muscles. The influence of temperature on the contractile properties of slow-twitch (S) MUs from soleus (SOL) muscles in rats was investigated under hypothermia (25 °C), normothermia (37 °C), and hyperthermia (41 °C). Hypothermia prolonged the twitch time parameters, decreased the rate of force development, increased the twitch-to-tetanus ratio, enhanced twitch force, and abolished post-tetanic depression. In contrast, hyperthermia did not alter twitch time parameters. Moreover, there was no effect on force despite the noted increase in post-tetanic depression and the twitch-to-tetanus ratio. Therefore, hypothermia induced more profound changes in S MUs compared with hyperthermia. The temperature effects in SOL MUs were compared to the effects previously reported for S MUs in the medial gastrocnemius (MG). The major differences between the S MUs of both muscles were the effects of temperature on twitch force, post-tetanic force modulation, twitch-to-tetanus ratio, and the slope of the force-frequency curve under hypothermia. Hyperthermia shortened twitch time parameters solely in the MG. In contrast, post-tetanic depression, twitch-to-tetanus ratio, and the slope of the force-frequency curve were influenced by hyperthermia only in SOL MUs. The different temperature effects of S MUs probably corresponded to differences in muscle architecture and their diverse functional tasks and enzyme activity. In summary, S MUs in SOL are more thermal-sensitive than their counterparts in MG.

## Introduction

Throughout their lives, humans are exposed to a wide range of environmental temperatures that may affect muscle performance^1^. Under extreme conditions, the body core temperature can vary from 25 °C (hypothermia) to 42 °C (hyperthermia)^2^. Daily fluctuations in various skeletal muscles range between 29 and 34 °C at rest and up to 41 °C during exhaustive exercises under normothermic conditions^3^. Additionally, environment-induced hyperthermia produces slightly different changes in contractile properties than exercise-induced hyperthermia^4^.

The contractile properties of fast-twitch muscles and fast-twitch motor units (MUs) are more temperature sensitive than those of slow-twitch muscles and slow-twitch (S) MUs, especially regarding force and force regulation parameters^5–7^. However, the shortening velocity and the rate of isometric tetanic force rise of slow-twitch muscles are more temperature sensitive^8^. On the other hand, the additional effects on time parameters of the twitch contraction and the rate of tetanic force development are similar for both types of muscles as well as for fast and slow motor unit types^5,6,9^. The variable temperature sensitivity of slow-twitch and fast-twitch muscles and motor units may be attributed to less developed sarcoplasmic reticulum and the lower Ca^2+^ ATPase activity in slow muscle fibers^10^. Hypothermia has a more profound effect on contractile muscle and MU properties than hyperthermia, predominantly prolonging time-related parameters and reducing the force regulation of MUs, especially fast-twitch MUs^7,8,11^. In contrast, twitch force, maximum tetanic force, and fatigue resistance are less sensitive to temperature changes. Furthermore, there are significant differences in the effects of temperature on fast-twitch fatigue-resistant (FR) and fast-twitch fatigable (FF) MUs, with FF MUs exhibiting higher sensitivity^6^.

The effect of temperature changes has been evaluated in various mammalian species, but only for one slow muscle: the soleus (SOL). For this muscle, intriguing interspecies differences were observed, such as the enhancement of the twitch force by hypothermia in mice, whereas it decreased in other species (rats and cats). These differences may be due to the varying composition of this muscle, which contains less than 60% slow-oxidative fibres in mice, 84% in rats, and nearly 100% in cats^12–14^. To date, the influence of temperature changes on the contractile properties of slow MUs has only been evaluated in rat medial gastrocnemius (MG) muscles^6^. Considering that the contractile properties of S MUs from rat MG and SOL muscles are considerably different regarding the force (higher in SOL) and twitch time parameters (longer in SOL) and the force regulation processes^15,16^, there is a gap in knowledge regarding the temperature sensitivity of S MUs in these two muscles.

In the present series of experiments, we investigated the influence of two extreme thermal conditions (hypothermia and hyperthermia) on S MUs of a typical S muscle in rats, the SOL, under the same experimental conditions as those used in recent studies on MUs of rat MG muscle. Based on these recently obtained data, we additionally compared the effect size (ES) of temperature between S MUs of the two hindlimb muscles: SOL and MG^6^. The comparison of these data is justified because the experimental series were conducted in the same laboratory using the same methods. We hypothesised that the contractile parameters of S MUs in SOL muscle will be affected mostly by hypothermia, and we expected considerable differences in temperature sensitivity between S MUs of SOL and MG muscle due to differences in muscle architecture^17^.

## Methods

Experiments were conducted on eight female Wistar rats (3–6 months old; body weight = 242.3 ± 7.3 g). Before the experiments, the animals were housed in standard laboratory cages (two per cage) with unrestricted access to standard laboratory food and water in a room set to a 12-h/12-h light/dark cycle, with humidity and temperature maintained at 55 ± 10% and 22 ± 2 °C, respectively. All procedures were approved by the Local Ethics Committee for Experiments on Animals in Poznan (approval no. 32/2019) and were performed according to the Polish Law on the Protection of Animals and European Union regulations, and Animal Research: Reporting of In Vivo Experiments.

Rats were deeply anesthetised with sodium pentobarbital (60 mg/kg, i.p., initial dose, supplemented as required). At the end of the experiment, the animals were euthanised by an overdose of sodium pentobarbital (180 mg/kg i.p.). The surgical preparation of rats was conducted in two phases. Initially, the distal part of the soleus muscle was partially isolated from surrounding tissue, preserving its innervation and blood supply, while other hind limb muscles were denervated by cutting the remaining branches of the sciatic nerve. Subsequently, a laminectomy was performed over the L2–S1 segments. Dorsal and ventral roots of the spinal nerves were cut close to the spinal cord. The animals were immobilized with steel clamps on the tibia, sacral bone, and the L1 vertebra. The isolated spinal cord, ventral and dorsal roots of spinal nerves were covered with warm paraffin oil in a small pool formed by the skin around the laminectomy^16,18,19^. During electrophysiological experiments, the animal’s hindlimb and SOL muscle were immersed in a metal pool filled with paraffin oil maintained at 25 ± 1 °C (hypothermia), 37 ± 1 °C (normothermia), or 41 ± 1 °C (hyperthermia). The animal’s rectal temperature was constantly measured, and internal muscle temperature was measured at the end of the experiments (Ella A/S; Hillerød, Denmark).

**Figure 1:**
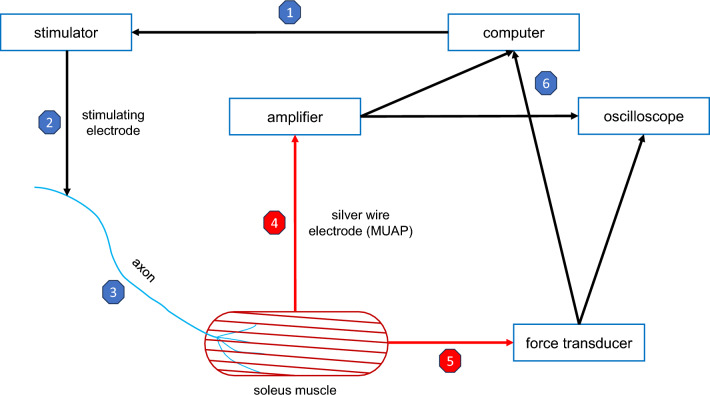
The scheme of the experimental setup for single motor unit recording. Initially, the computer triggers the stimulator (1) which via electrode (2) delivers the stimulus to the axon in a fragment of ventral root and the action potential conducted within the nerve (3) evoke the motor unit action potential (MUAP) and a twitch contraction in soleus. The MUAP is collected with silver wire bipolar electrode and amplified (4). Simultaneously, motor unit contraction is measured by a force transducer (5). Both the MUAP and force are recorded and saved on the computer hard drive, as well as presented in real-time on the oscilloscope (6).

The soleus was linked to an inductive force transducer via the distal tendon. Isometric contractions were measured in a muscle stretched up to a passive tension of 40 mN, ensuring the assessment of the peak contractile force of MUs^18^. To elicit the evoked activity of individual motor units, L5 or L4 ventral roots were meticulously divided into fine axon bundles, subsequently positioned on a silver wire electrode, and subjected to electrical stimulation using 0.1 ms rectangular pulses with amplitudes up to 0.5 V. The electrical pulses were generated by a dual-channel square pulse stimulator (Grass Instrument Company; Grass Instruments S88 Dual Output Square Pulse Stimulator; West Warwick, RI, USA). All force recordings were collected using a 12-bit analogue-to-digital converter (RTI-800; Analog Devices; Wilmington, MA, USA) at a sampling rate of 10 kHz, and data were stored for further analyses (Fig. 1).

The following experimental protocol was performed:

1. Five stimuli at 1 Hz (five twitches recorded)
2. Train of stimuli at 40 Hz and 500 ms duration (unfused tetanus recorded)
3. Train of stimuli at 150 Hz and 300 ms duration (fused tetanus recorded)
4. Five stimuli at 1 Hz
5. Trains of stimuli at 5, 10, 15, 20, 25, 30, 35, 40, 50, 60, 75, 100, and 150 Hz and for 500 ms duration, with 10-s intervals between trains (twitches, unfused and fused tetani recorded)
6. Five stimuli at 1 Hz
7. Fatigue test (14 stimuli at 40 Hz repeated every second for 3 min)^20,21^; due to prolonged relaxation in hypothermia, an additional 1-s break was taken every 10 tetani to control the fused tetanus release
8. Five stimuli at 1 Hz

Ten-second time intervals were applied between consecutive steps of the protocol.

Twitches were averaged for steps 1, 4, 6, and 8. Basic contractile properties were determined for all MUs. The twitch force and maximum tetanus force were measured at the peak of twitch and tetanus amplitude, respectively; the twitch-to-tetanus ratio was then calculated. The unfused tetanus (40 Hz) was recorded to observe sag^22^. The contraction time was measured from the beginning of the twitch to the peak of the force record, while the half-relaxation time was measured from the peak to the moment when the force decreased to half of the peak value. The rate of force development (RFD) was calculated from the onset (the point when a recording exceeded the noise level) to half-maximal force. Post-tetanic depression (TwD) was calculated as the ratio of twitch forces in steps 4 and 1. The force-frequency relationship was determined for the recordings in step 5. During the fatigue test, the resistance index (RI) was calculated as the ratio of the force generated 120 s after the initial force^23^. The peak forces of successive tetani during the fatigue test were measured and are presented as the percentage of force relative to the initial peak force on a time scale.

All data are expressed as mean ± standard deviation (SD), with the median values provided in parentheses in the tables. The normality of the interval scale data distribution was assessed using the Shapiro–Wilk test, and the homogeneity of variance of the data was assessed using Levene’s test. Due to the normality of the distribution of our data, all comparisons used a one-way analysis of variance (ANOVA) with a post-hoc Tukey test of unequal sample size or a Kruskal–Wallis test with a multiple of the mean rank. Force profiles from the fatigue test were compared using repeated measures ANOVA. The ES measured as Cohen’s d (referred to as ‘d’) was used to compare changes in contractile properties between groups with different sample sizes.

All statistical tests were performed using RStudio 4.0.2 (RStudio, PBC; Boston, MA, USA) and Statistica 13.3 (StatSoft; Kraków, Poland).

### Ethics statement

We confirm that we have read the Journal’s position on issues involved in ethical publication and affirm that this manuscript is consistent with those guidelines.

## Results

**Figure 2:**
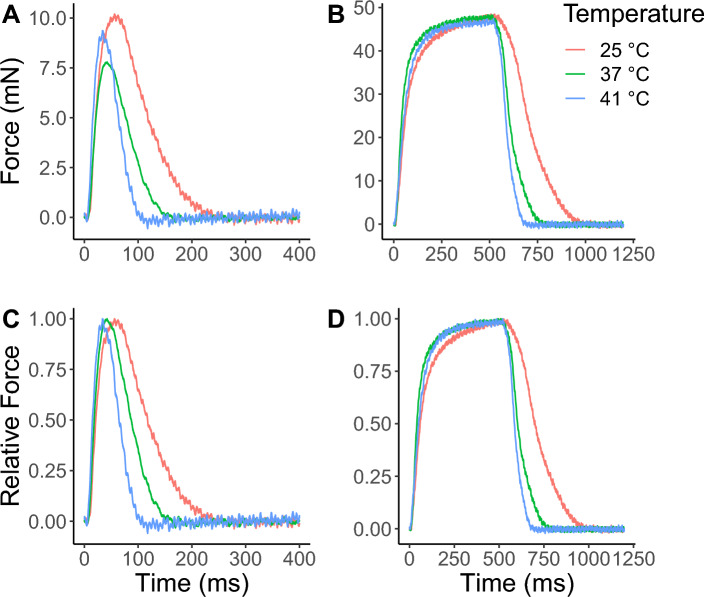
The representative myograph of slow-twitch motor units (S MUs) of rat soleus (SOL) at 25 ± 1 °C (hypothermia, red), 37 ± 1 °C (normothermia, green), or 41 ± 1 °C (hyperthermia, blue). (**A**) Twitch force. (**B**) Fused tetanic force. (C) Relative twitch force. (D) Relative fused tetanic force.

We evaluated the mechanical properties of 99 S MUs divided into three experimental groups: hypothermia (n = 48; from 4 rats), normothermia (n = 24; from 2 rats), and hyperthermia (n = 27; from 2 rats). The rats’ mean measured core temperatures under each condition were 29.2 ± 0.8 °C, 35.7 ± 1.1 °C, and 38.6 ± 1.4 °C, respectively, and their mean muscle temperatures were 26.7 ± 0.2 °C, 34.3 ± 0.7 °C, and 38.3 ± 0.1 °C, respectively (Fig. 2).

**Figure 3:**
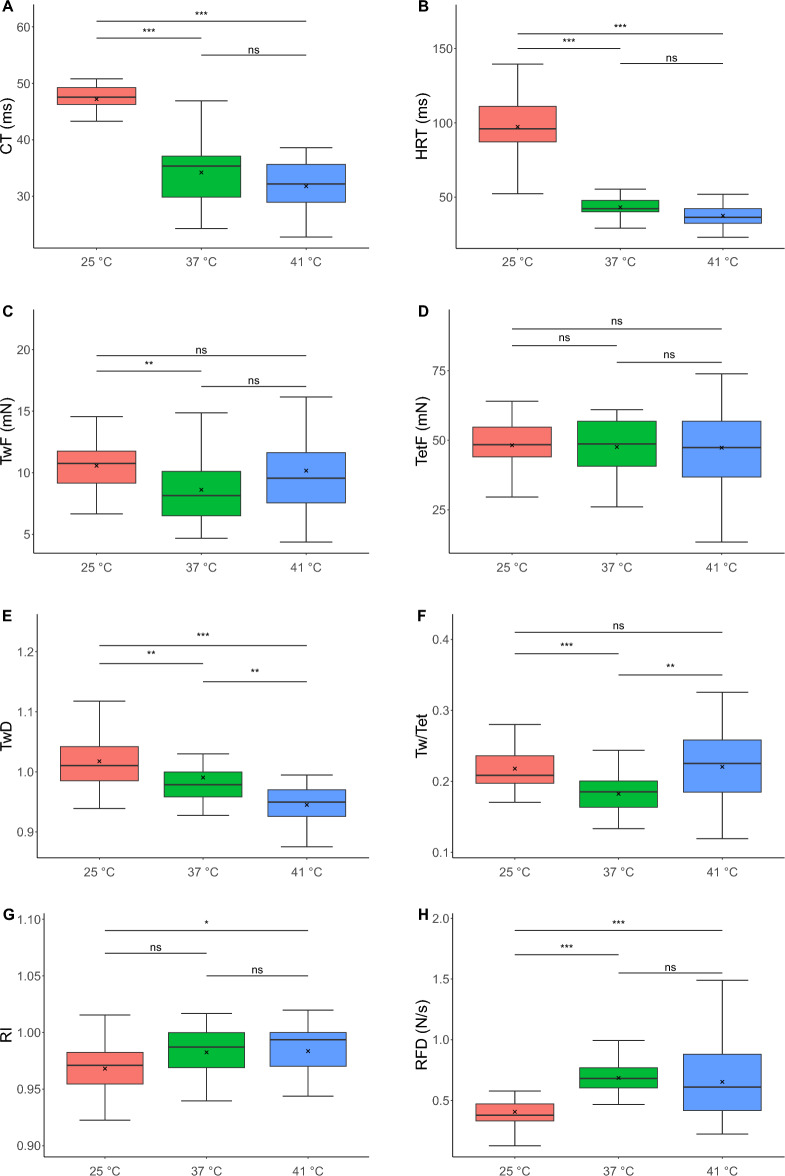
Contractile parameters of slow-twitch motor units (S MUs) of rat soleus (SOL) at 25 ± 1 °C (hypothermia, red), 37 ± 1 °C (normothermia, green), or 41 ± 1 °C (hyperthermia, blue). (**A**) Contraction time. (**B**) Half-relaxation time. (**C**) Twitch force. (**D**) Fused tetanic force. (**E**) Post-tetanic depression of twitch. (**F**) Twitch-to-tetanus ratio. (**G**) Resistance index. (**H**) Rate of force development up to 50% of maximum force. Boxplot showing the median (horizontal black line inside the box) and mean (black cross) values and the interquartile range (IQR; 25–75%) with minimum (Q_1_ − 1.5 × IQR) and maximum (Q_3_ − 1.5 × IQR) values. Key: ns, nonsignificant difference; *, *p* < 0.05; **, *p* < 0.01; ***, *p* < 0.001.

**Figure 4:**
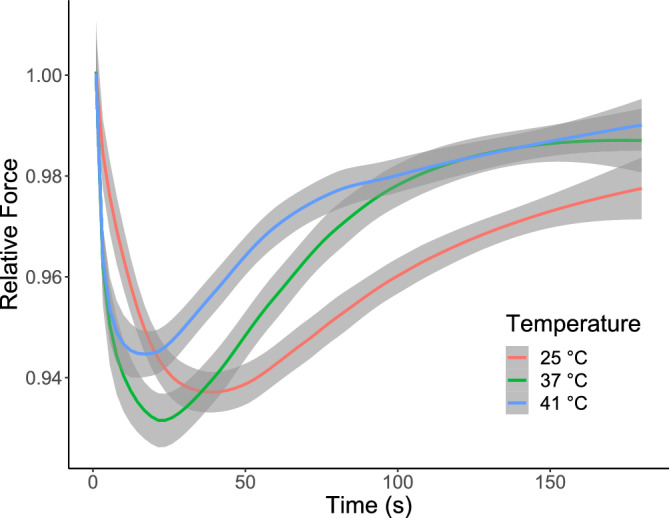
Relative forces produced by the S MUs of rat SOL during the fatigue test at 25 ± 1 °C (hypothermia), 37 ± 1 °C (normothermia), and 41 ± 1 °C (hyperthermia). The force of 1.0 corresponds to the highest force recorded at the beginning of the fatigue test. The grey area around the line demarcates the 95% confidence interval.

**Figure 5:**
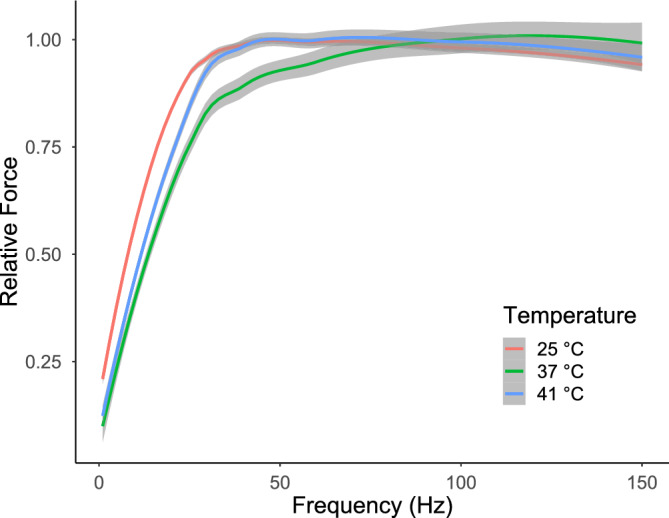
Force-frequency curves of the S MUs of rat SOL at 25 ± 1 °C (hypothermia), 37 ± 1 °C (normothermia), and 41 ± 1 °C (hyperthermia). A relative force of 1.0 is the maximum tetanus force. The grey area around the line demarcates the 95% confidence interval.

The effects of hypothermia and hyperthermia on MU properties were investigated and compared to normothermia. The ES from this study was then compared to our previous findings from MG muscle^6^. Hypothermia significantly increased both time-related parameters: contraction time (*d* = − 3.26, 95% confidence interval [CI − 3.98, − 2.53]; Fig. 3A) and half-relaxation time (*d* = − 2.62 [− 3.27, − 1.96]; Fig. 3B). In contrast, force-related parameters exhibited minimal temperature sensitivity. The twitch under hypothermia revealed ‘cooling potentiation’ and was stronger (*d* = − 0.82 [− 1.33, − 0.31]; Fig. 3C); however, the fused tetanic force did not change (Fig. 3D). Interestingly, the post-tetanic depression of twitch force observed during normothermia was abolished (*d* = − 0.56 [− 1.06, − 0.06]; Fig. 3E). Moreover, hypothermia significantly decreased the RFD (*d* = 1.67 [1.10, 2.24]; Fig. 3H). Although the RI only exhibited some tendency to decrease under low-temperature conditions (*p* = 0.07, *d* = 0.33 [− 0.18, 0.85]; Fig. 3G), hypothermia modified the profiles of tetanic force changes during the fatigue tests. Significant differences were observed within 10–20 s (0.000008 < *p* < 0.000093), 60–110 s (0.00742 < *p* < 0.045488), and at 130 s (*p* = 0.024841; Fig. 4). Furthermore, hypothermia increased the twitch-to-tetanus ratio (Fig. 3F) and altered the force-frequency curve, which was shifted towards lower stimulation rates; however, the slope of the curve did not change significantly under hypothermic conditions (Fig. 5). The stimulation frequency needed to produce 75% of the maximum force was decreased during hypothermia (*d* = 1.73 [1.16, 2.30]; Table 1).

**Table 1:** Properties of the force-frequency relationships of MUs in rat SOL at three temperatures.

	Temperature			*p*
	Hypothermia	Normothermia	Hyperthermia	
	(25 °C)	(37 °C)	(41 °C)	
Freq 75% *(Hz)*	14.3 ± 2.4 (14.7)^b,c^	23.0 ± 8.0 (22.7)^a^	18.4 ± 17.8 (3.1)^a^	< 0.0001^§^
Slope *(%/Hz)*	2.7 ± 0.8 (2.5)^c^	2.5 ± 1.5 (2.0)^c^	3.5 ± 1.0 (3.2)^a,b^	< 0.001^§^

The mean ± SD and median values. Key: Freq 75%, the stimulation frequency at 75% of the maximum force; Slope, the slope of the curve at 75% of the maximum force; §, Kruskal–Wallis rank test; #, one-way ANOVA; a, significantly different from 25 °C; b, significantly different from 37 °C; c, significantly different from 41 °C.

Hyperthermia mostly had the opposite effect of hypothermia; however, many of these differences were not statistically significant. There were no changes in the contraction or half-relaxation times (Fig. 3A,B). Likewise, twitch force, tetanus force, and RFD were not altered under hyperthermic conditions (Fig. 3C,D,H); however, post-tetanic depression of twitch was augmented (*d* = − 0.86 [− 1.43, − 0.29]; Fig. 3E). RI was unchanged under hyperthermic conditions (Fig. 3G). Hyperthermia moderately influenced the profile of force changes during the fatigue test; higher relative forces were noted from 30 to 60 s (0.001183 < *p* < 0.034204; Fig. 4). Interestingly, hyperthermia increased the twitch-to-tetanus ratio (*d* = − 0.86 [− 1.43, − 0.29]; Fig. 3F) and the slope of the force-frequency curve (*d* = 0.77 [0.20, 1.34]; Fig. 5). In contrast, the stimulation frequency needed to elicit 75% of the maximum force was not affected by hyperthermia (Table 1). Surprisingly, the force-frequency curve, as in hypothermia, shifted towards lower frequencies, and MUs reached maximal force at approximately 50 Hz, whereas the force decreased at higher stimulation frequencies (Fig. 5).

**Figure 6:**
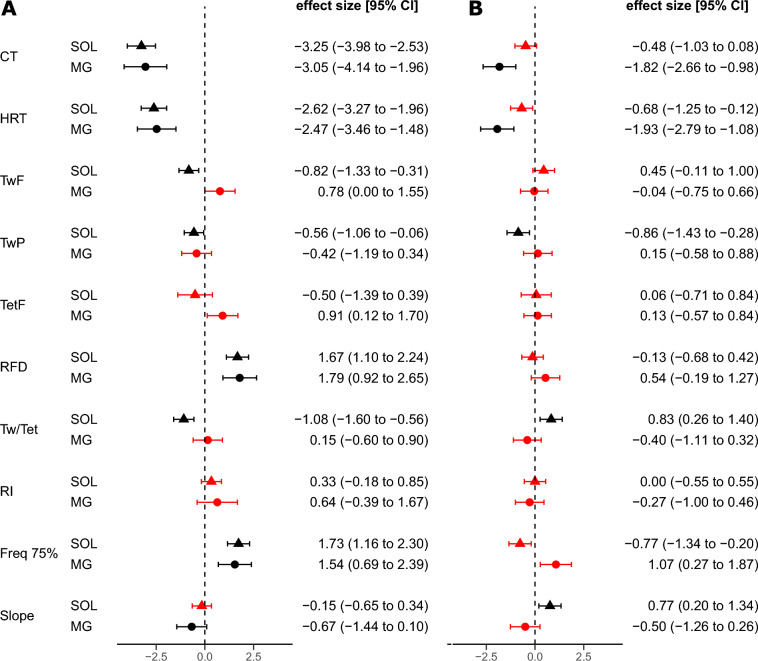
The effect size and 95% confidence interval values presented for S MUs in SOL from this study and MG^6^ at (**A**) 25 ± 1 °C (hypothermia) vs 37 ± 1 °C (normothermia) and (**B**) 37 ± 1 °C (normothermia) vs 41 ± 1 °C (hyperthermia). Triangle represents SOL, circle represents MG; red (SOL and MG) colour indicates no significant differences between groups for a given muscle; black (SOL and MG) colour indicates the presence of significant differences between groups. Abbreviations as in Table 2.

The effects of temperature on the contractile properties of S MUs in MG and SOL under the conditions of hypothermia and hyperthermia were compared based on recently published data^6^ (Fig. 6) and the applied effect size indicated how hypo- and hyperthermia influenced these parameters (i.e. the raw values of force and time parameters were not directly compared between the S motor units from MG and SOL). The notable changes in twitch force, post-tetanic depression of twitch, and twitch-to-tetanus ratio observed in SOL muscle under hypothermic conditions were not observed in MG muscle. For the two muscles the direction of changes in twitch force, tetanus force, and the twitch-to-tetanus ratio was inverse in hypothermia. Furthermore, the slope of the force-frequency curve was significantly decreased in S MUs in MG (Fig. 6A).

In both SOL and MG muscles, hyperthermia had less of an impact on the contractile properties of MUs than hypothermia. However, hyperthermia altered twitch time parameters, such as contraction time and half-relaxation time, in S MUs in MG, but not in SOL (Fig. 6). In contrast, increases in the post-tetanic depression of twitch, twitch-to-tetanus ratio, and the force-frequency slope were observed solely in SOL. The twitch-to-tetanus ratio, the stimulation frequency at 75% of the tetanic force, and the slope of the force-frequency curve tended to change in opposite directions for SOL and MG under hyperthermia condition (Fig. 6B).

## Discussion

This study was the first to document the influence of temperature on the contractile properties and force regulation of functionally isolated S MUs of the SOL muscle. As hypothesised, the effects of hypothermia were greater than those of hyperthermia. Moreover, the time twitch parameters and RFD were more impacted by hypothermia; in contrast, hyperthermia did not significantly affect these parameters. Interestingly, hypothermia negated the effect of post-tetanic depression, which was observed in the majority of MUs of SOL in normothermia. On the other hand, hyperthermia deepened this phenomenon. As far as we know, the mechanisms underlying the post-tetanic twitch depression in slow MUs, as well as slow-twitch muscles, remain unknown. Nevertheless, hypothermia lessened these mechanisms, while hyperthermia intensified them. These effects could be associated with changes in myofilament sensitivity to calcium or the reaccumulation of calcium after previous activity, as well as impaired of membrane excitability^24–26^. Amobi and Smith demonstrated that post-tetanic twitch depression in smooth muscles (vas/ductus deferens) has an intrinsic presynaptic origin^27^. However, in cat soleus the amplitude of EMG recorded in most cases remained within ± 5% of the pretetanic value^24^. Both extreme thermal conditions increased the twitch-to-tetanus ratio; however, the frequency corresponding to 75% of the maximum force was increased in hypothermia while the force-frequency curve became steeper in hyperthermia. These observations are partially compatible with earlier studies on whole slow muscles of different species^7,11,14^. Prolongation of the twitch time parameters was noted in these studies; however, ‘twitch cooling potentiation’ was only reported in mouse SOL. The causes of variable effects of hypothermia may be associated with the diverse composition of muscle (the lowest participation of slow muscle fibres in mouse SOL). Our previous research partially confirms this thesis, as this phenomenon was only observed in fast-twitch FR MUs, however S MUs in SOL also showed ‘twitch cooling potentation’^12,13^. The mechanism remains unknown. While fatigue resistance in S MUs was invariant under the examined temperatures (Table 2), the course of initial changes in the force of tetanic contractions during the fatigue test revealed temperature dependence (slower in hypothermia, Fig. 4)^28^. The literature data have reported the decreased fatigability of SOL muscle at 25–30 °C compared with 20 °C and 35–40 °C although fatigue test protocols and the type of contraction varied between studies^7,29,30^. Our findings stem from a multitude of thermosensitive processes, encompassing axon conductance, end-plate transmission, and metabolic and molecular processes within muscle fibres^5^. In lower temperatures, voltage-gated sodium channels in nerves, endplates, and muscles exhibit prolonged openness^31,32^, leading to increased amplitude and duration of action potentials. Alterations in sodium channel kinetics, induced by slower depolarisation in nerve and muscle membranes, disturb conduction velocity and impact the resting membrane potential^33^. Conversely, heating exerts the opposite effect^32^. Furthermore, temperature-dependent variations in myofibrillar Ca^2+^ sensitivity and the reabsorption rate of Ca^2+^ ions by the sarcoplasmic reticulum contribute to these modifications^34,35^. These temperature-induced changes reshape the characteristics of twitch kinetics, especially time-related parameters. The tetanic contraction is an effect of summation of twitch-shape responses to individual activations. Consequently, these alterations influence the processes of force regulation and modulate the force-frequency relationship. Finally, it is worth stressing that for some MUs, especially in hypothermia, at high stimulation frequencies (100–150 Hz), smaller tetanic force was recorded in relation to lower frequencies (50–75 Hz). This effect seems to be related to disorders in the neuromuscular transmission^36^. On the other hand, the force-frequency curve shifted left in hypo- and hyperthermia compared to normothermia. This suggests that impaired sarcoplasmic reticulum Ca^2+^ release and myofibrillar Ca^2+^ sensitivity as well as increased twitch-to-tetanus ratio in both examined temperatures may be potential causes of the force-frequency shift^37,38^.

**Table 2:** The contractile properties of S MUs in rat SOL at three temperatures.

	Temperature			*p*
	Hypothermia	Normothermia	Hyperthermia	
	(25 °C)	(37 °C)	(41 °C)	
CT *(ms)*	47.2 ± 2.7 (47.5)^b,c^	34.2 ± 5.8 (35.4)^a^	31.8 ± 4.5 (32.2)^a^	< 0.0001^#^
HRT *(ms)*	97.4 ± 24.6 (90.1)^b,c^	43.2 ± 8.6 (42.2)^a,c^	37.5 ± 8.0 (36.4)^a,b^	< 0.0001^§^
TwF *(mN)*	10.6 ± 2.3 (10.8)^b^	8.6 ± 2.5 (8.2)^a^	10.2 ± 4.1 (9.6)	< 0.05^#^
TwD	1.02 ± 0.05 (1.01)^b,c^	0.99 ± 0.06 (0.98)^a,c^	0.95 ± 0.03 (0.95)^a,b^	< 0.001^#^
TetF *(mN)*	48.2 ± 9.4 (48.4)	47.6 ± 10.6 (48.7)	47.3 ± 16.3 (47.4)	n.s.*
RFD *(N/s)*	0.41 ± 0.14 (0.38)^b,c^	0.69 ± 0.21 (0.68)^a^	0.65 ± 0.29 (0.61)^a^	< 0.0001^§^
Tw/Tet	0.22 ± 0.04 (0.23)^b^	0.18 ± 0.03 (0.19)^a,c^	0.22 ± 0.06 (0.23)^b^	< 0.001^#^
RI	0.97 ± 0.03 (0.97)	0.98 ± 0.03 (0.99)	0.98 ± 0.03 (0.99)	< 0.05^#^

The mean ± SD and median values of basic contractile properties. Key: CT, contraction time; HRT, half-relaxation time; TwF, twitch force; TwD, post-tetanic depression of twitch; TetF, tetanic force; RFD, rate of force development; Tw/Tet, twitch-to-tetanus ratio; RI, resistance index; §, Kruskal–Wallis rank test; #, one-way ANOVA; a, significantly different from 25 °C; b, significantly different from 37 °C; c, significantly different from 41 °C; n.s., non-significant.

The advantage of the present research is the comparison of the temperature sensitivity of S MUs from SOL and recently studied MG muscles. The MG and SOL are parts of the triceps surae muscle, which inserts into the calcaneus and is active during plantarflexion. S MUs in both muscles are also involved in antigravity tasks; however, S MUs comprise about 10% of the MU population in MG and almost 100% in SOL. However, the contractile properties of the S MUs in these two muscles reveal considerable differences. The most important differences are the longer contraction and relaxation times and the higher twitch force in SOL^16^. These differences are likely due to the differences in muscle architecture (pennation angle, muscle fibre/muscle length ratio), innervation ratio, muscle fibre diameter, density within an MU territory, or the location of these two muscles (deeper for SOL, superficial for MG)^39^. The fibres of rat MG muscles are shorter, have larger diameters and physiological cross-sectional area, and have lower fibre-muscle length ratios than SOL muscles. Moreover, the pennation angle, which is important for the force transmission, is almost four times larger in MG (14.0° ± 3.9°) compared with SOL (3.9° ± 2.4°). When individual motor units contract in pennated muscles a part of produced force is used to rotate muscle and stretch non-contacting muscle fibres and this effect was observed as deformation of the muscle surface (mechanomyogram)^40^. The differences between muscle architecture (semipennate MG and pennate SOL) should also be considered^17,41^. Additionally, the innervation ratio for slow MUs in SOL is considerably higher in relation to MG (ranges 84–178 and 41–80, respectively)^42,43^. The twitch force (directly related to the innervation ratio) even within motor units of the same type correlates with the twitch-to-tetanus ratio^44^. However, there is no literature data indicating that these differences in muscle structure may influence sensibility to changes in muscle temperature. Furthermore, the contractile properties of MUs depend on their activity level. Hennig and Lømo^45^ reported that during normal daily activity, rat hindlimb MUs exhibit three distinct patterns of activity: long-lasting tonic activity (approximately 5–8 h of total daily activity, observed for MUs in SOL), frequent activity (20–90 min daily, observed in extensor digitorum longus), and sporadic activity (from 30 s to 3 min daily, observed in extensor digitorum longus). These three distinct periods of activity were characteristic of S, FR, and FF MUs, respectively. There are no data describing the activity of MUs in rat MG muscle; however, it is possible that their S MU activity time is divergent in relation to SOL MU activity time. Perhaps, because of these anatomical and biomechanical differences, force parameters of S MUs in SOL are more hypothermia-dependent than S MUs in MG. Furthermore, the important force regulation parameter, the twitch-to-tetanus ratio, was increased in S MUs of SOL, but not in S MUs of MG. The time parameters of S MUs in SOL were invariant under hyperthermia; however, they were altered in the S MUs of MG. In addition, the RI in S MUs from both SOL and MG lacked sensitivity to thermal conditions, but the fatigue test course of S MUs in SOL was affected by both hypothermia and hyperthermia^6^.

The cause of the observed temperature sensitivity may be related to the high thermosensitivity of Ca^2+^ ATPase activity, which conditions the contraction time^35,46^. It may also be influenced by changes in the activity level of other enzymes, for instance, acetylcholinesterase (reduced by hypothermia), and the slower opening/closing of Na^+^ ion channels at lower temperature. These effects may vary in intensity among various muscles^31,32^.

In conclusion, the investigation of the contractile properties of S MUs in rat SOL muscle at two examined temperatures compared with normal temperature revealed that these properties were more sensitive to hypothermia. The most sensitive properties were twitch time parameters and force regulation by changes in the stimulation frequency. A comparison of the present data to the recently reported effects of temperature changes on S MUs in rat MG revealed significant differences that were most likely related to differences in muscle structure, location, enzyme activity, and/or ion channel kinetics. Therefore, S MUs in SOL were more thermal-sensitive than their counterparts in MG.

## Data Availability

The data that support the findings of this study are available from the corresponding author upon reasonable request.

## Abbreviations

ANOVA: Analysis of variance
CI: Confidence interval
CT: Contraction time
ES: Effect size
FF: Fast-twitch fatigable
FR: Fast-twitch fatigue-resistant
Freq 75%: The stimulation frequency at 75% of the maximum force
HRT: Half-relaxation time
IQR: Interquartile range
MG: Medial gastrocnemius
MUs: Motor units
RFD: Rate of force development
RI: Resistance index
S: Slow-twitch
SD: Standard deviation
Slope: The slope of the curve at 75% of the maximum force
SOL: Soleus
TetF: Fused tetanic force
TwD: Post-tetanic depression of twitch
TwF: Twitch force
Tw/Tet: Twitch-to-tetanus ratio
